# Development of novel *Aotus nancymaae* non-human primate model for the evaluation of *Plasmodium vivax* blood stage vaccines and immunoprophylactics

**DOI:** 10.1186/s12936-026-05960-7

**Published:** 2026-06-03

**Authors:** Julio A. Ventocilla, L. Lorena Tapia, Fredy E. Villena, Diana Cedamanos, Jessica Buchta, Danielle Pannebaker, Hugo O. Valdivia, Karli R. Redinger, Jürgen Bosch, Lenore Carias, Berlin Londono-Renteria, Christopher King, Brandon K. Wilder

**Affiliations:** 1 Culmen Inc, Alexandria, VA USA; 2 U.S. Naval Medical Research Unit SOUTH (NAMRU SOUTH), Lima, Peru; 3 Case Western Reserve University, Cleveland, OH USA; 4 Oregon Health & Science University, Portland, OR USA; 5 Universidade Federale de Minas Gerais, Belo Horizonte, Brazil; 6 Veterans Affairs Medical Center, Cleveland, OH USA; 7 InterRayBio, LLC, Cleveland, USA

**Keywords:** Aot*us nancymaae*, *Plasmodium vivax*, Blood stage model and monoclonal antibodies

## Abstract

**Background:**

Few well-established in vivo animal models reflect the complexity of human malaria infection and immunity, and even fewer have been predictive of the potential of malaria vaccines or prophylactic therapies. Here, we characterized and optimized an *Aotus nancymaae* non-human primate (NHP) model of *Plasmodium vivax* (Pv) blood stage infection to enable the testing of novel vaccines and immunoprophylactics.

**Methods:**

Using direct blood stage infection, we selected Vietnam-IV as the most virulent *P. vivax* strain from seven monkey adapted strains. We next determined the infectious dose with the lowest variability at doses of 0.3 (n = 3), 1.0 (n = 6) and 2.5 (n = 3) × 10^6 infected red blood cells (iRBC) in spleen-intact *A. nancymaae* non-human primates (NHPs). In addition, complete blood count (CBC) was measured during blood stage infection. Genomic sequencing was performed for *P. vivax* Vietnam-IV candidate Duffy Binding Protein-II. Finally, we evaluated the efficacy of a monoclonal antibody (mAb 099100) against DBP-II to protect against blood infection challenge.

**Results:**

The *P. vivax* Vietnam-IV monkey adapted strain at dose of 2.5 × 10^6 gave infection kinetics with the lowest coefficient variation (15–23%). CBC results at a low (0.3 × 10^6) and high (2.5 × 10^6) dose showed a significant increase in lymphocytes (5.0–5.3 to 7.0–8.5 10^9/l) and a decrease of neutrophils (1.9–3.0 to 0.6–1.2) and platelets (413–458 to 23–68 10^9/l) during acute infection. In a pilot study testing the anti-DBPII mAb 099100, we observed that 1 of 3 monkeys was partially protected against *P. vivax* as measured by need for treatment intervention (compared with 0 out of 3 monkeys in the control group). *P. vivax* Vietnam-IV genomic data showed that line was a single clone with a single gene copy of blood stage *PvDBPII* with single nucleotide polymorphisms outside mAb binding sites.

**Conclusions:**

Our study showed a *P. vivax* blood stage *A. nancymaae* model with well-characterized clinical, hematological and serological parameters during the course of infection. The challenge appeared stringent and suggests a high titer or high potency of mAb is needed for protection. This model can set the foundation for the evaluation of emerging *P. vivax* blood stage vaccines, monoclonal antibodies, anti-malarial drugs and prophylactics.

**Supplementary Information:**

The online version contains supplementary material available at 10.1186/s12936-026-05960-7.

## Introduction

Malaria is caused by infection with Plasmodium parasites and is endemic in regions that encompass about a third of the world’s population, including the Middle East, Africa, Southeast Asia, and South America. The global annual incidence of malaria in 2023 was approximately 250 million cases, resulting in over half a million deaths, mostly among young children in sub-Saharan Africa [1] Conversely, populations outside endemic areas, such as U.S. military personnel and civilian travelers, face a particularly high risk of malaria during both long- and short-term visits to highly endemic regions. While most malaria deaths are caused by *P. falciparum*, the second most common parasite, *P. vivax*, can also cause mild to severe symptoms including fever, chills, headache, and even death if not diagnosed and treated quickly. These symptoms can significantly impair the performance of military and civilian personnel during critical operations [2, 3]. Additionally, *P. vivax* has a broader geographic distribution than *P. falciparum*, causing up to 14 million clinical cases each year in endemic areas including Asia and South.

Although malaria prevention during military deployment or travel can be achieved by chemoprophylaxis with daily doxycycline or weekly mefloquine, compliance is very low due to the need for repeated oral administration and side effects [4]. More recently, tafenoquine has demonstrated higher adherence levels in military and civilian populations using single dose administration, reducing the risk of *P. vivax* recurrence by activation of dormant parasites in liver [5]. While chemoprophylaxis and other strategies such as the use of insecticide-treated bed nets and active surveillance have roughly halved disease burden in the twenty-first century, malaria control programs struggle to make sustained gains especially in highly endemic areas. This highlights the need for new strategies to fight malaria and to reduce the risk of infection in endemic and non-endemic malaria populations [1, 4].

Over the last decade, *P. falciparum* malaria researchers reported unprecedented advances in discovering and evaluating vaccines. In 2022, the World Health Organization (WHO) approved the first malaria vaccine for *P. falciparum* (RTS,S/AS01), which targets the parasite as it enters the skin and migrates through the blood to the liver (i.e., the “pre-erythrocytic” or PE stages) by inducing antibodies that recognize a major parasite antigen, the surface circumsporozoite protein (CSP). This vaccine prevents 20–36% of severe disease over four years [6, 7]. In 2023, a second vaccine (R21/Matrix-M) was recommended for use by WHO. R21 uses a different adjuvant but, like RTS,S/AS01, targets CSP but with an increased ratio of CSP protein (4 times more) on the antigen. R21 has 68–82% efficacy over 12 months in clinical Phases I-III [7–9]. Both vaccines suffer the limitation that if the parasite escapes anti-CSP antibodies and establishes infection in the liver, the vaccine-induced antibodies are no longer useful. This is because during the weeklong liver stage, the parasite replicates and transforms into “merozoites” that are released into the blood where they cyclically infect red blood cells to cause symptomatic infection and no longer express CSP.

This blood stage is also an appealing target for vaccines and preventive therapies. For *P. falciparum*, many potential vaccine targets such as the merozoite surface protein (MSP) family, erythrocyte binding antigen (EBA) family, members of the complex Rh5, CyRPA and Ripr (RCR), apical membrane antigen 1-rhoptry neck protein 2 (AMA1-RON2) and others have been evaluated with varying success [10–12]. The most successful has been *Pf* Rh5/Matrix-M which recently demonstrated 55% efficacy against clinical malaria in a field trial with young children [13]. Of note, advancement of this and other blood stage *P. falciparum* vaccine candidates has been accelerated by the ability to culture *Pf* blood stages in vitro and the ability to transmit these parasites to mosquitos to enable the study of the sporozoite and liver stages to some extent in vitro and in vivo. The growth inhibition assay (GIA), developed by the U.S. National Institutes of Health (NIH), has been used as a tool to measure inhibition of blood stage invasion by IgG antibodies against merozoites, similar to a viral neutralization assay [14–16]. In addition, *Aotus nancymaae* non-human primates can be infected with the blood stages of *Pf*, which we and others have used as a stringent model predictive of human vaccine efficacy [17–21].

In contrast to *P. falciparum*, the lack of robust in vitro and in vivo models of *P. vivax* has created a bottleneck for evaluating novel blood stage vaccines and a paucity of candidates in the pipeline. In vitro* P. vivax* culture has been attempted for decades with limited success, with only a short invasion assay that depends on using field isolates in culture with constant supplementation with fresh reticulocytes. Other testing methods include the use of transgenic *P. knowlesi* parasites modified to express *P. vivax* genes [22–24] and closely genetically related *P. cynomolgi*, both utilizing in rhesus non-human primates [25–27]. Human liver-chimeric mice allow for *P. vivax* sporozoite infection but lack any adaptive immune system—precluding active immunization—and need a continuous source of reticulocytes for even a modest blood stage infection [28].

Few *P. vivax* blood stage vaccine candidates have been well-characterized in immunocompetent animal models using non-human primates (NHP) and even fewer in clinical studies. Early studies showed that the new world *Aotus* genus are susceptible to blood *P. vivax* infection, and have been used for characterization of immunogenicity and efficacy of blood stage vaccine targets as MSP and RAP2 proteins although low and variable parasitemia in spleen-intact animals was limiting [29–31]. More recently, *Aotus lemurinus* NHPs have been used as a valuable tool for down selection of novel vaccines in pre-clinical studies. Plasmid DNA and adenovirus vectors targeting *P. vivax* AMA1 and MSP1-42 antigens showed modest delays of prepatent parasitemia period after *P. vivax* challenge with the Sal-I strain in spleen-intact *Aotus lemurinus* model [32].

Controlled human malaria infection (CHMI) using *P. vivax* blood stages has been utilized more recently for evaluation of blood stage vaccine candidates. A novel *P. vivax* W1 strain from Thailand has been optimized for use in CHMI, showing safety and robust infectivity. In addition, full genomic characterization of *P. vivax* W1 strain is available for better design of novel drug and vaccines against *P. vivax* [33]. More recently, this model was used to test the efficacy of two *P. vivax* vaccines (Chimpanzee Adenovirus 63/modified vaccinia virus Ankara versus protein and Matrix-M adjuvant formulation) targeting Duffy-binding protein region II (DBP-II) in Phase I/IIa vaccine efficacy trials. Results showed a delay in multiplication rate in human blood for the DBPII/Matrix-M group given a delayed third dose [34]. This first evidence of vaccine efficacy against *P. vivax* infection in humans encourages the evaluation of novel vaccine targets.

Here, we aimed to fill a gap in the *P. vivax* preclinical pipeline with a well-characterized, immune competent animal model capable of supporting the down selection of novel *P. vivax* blood stage vaccine candidates prior to human clinical trials. To this end, we tested multiple strains of *P. vivax* and optimized a reliable and predictable *P. vivax* blood stage infection model using *Aotus nancymaae* NHPs.

## Materials and methods

### *Aotus nancymaae* monkeys

The experiments reported herein were conducted in compliance with the Animal Welfare Act and in accordance with principles set forth in the “Guide for the Care and Use of Laboratory Animals”, Institute of Laboratory Animals Resources, National Research Council, National Academy Press, 2011. All animals were bred in captivity and included in NAMRU SOUTH Institutional Animal Care and Use Committee (IACUC)-approved protocols. In addition, these animal studies were approved by the Peruvian government agency of “Servicio Nacional Forestal y de Fauna Silvestre” (SERFOR). All approvals and protocols reviews were obtained before the study was performed. *Aotus nancymaae* NHPs were between 1–3 years old and weighed over 700 g in this study.

### *P. vivax* monkey adapted strains

William E. Collins, a malariologist researcher from the U.S. Centers for Disease Control and Prevention (CDC), conducted studies adapting *P. vivax* strains from humans to blood stage in *Aotus* NHPs, producing a generous cryobank for basic malaria research between 1970 and 1990 which were shared with collaborators worldwide including the National Institute of Allergy and Infectious Diseases (NIAID) [35–37]. For this project, cryopreserved monkey *P. vivax* adapted strains including Vietnam-IV, AMRU-I, Brazil-I, Sal-I, ONG, Apastepeque, and Chesson were generously provided by Dr. Thomas Wellems from the Laboratory of Malaria and Vector Research at NIAID.

### *P. vivax* infection and monitoring

To determine the most virulent strain measured by parasitemia levels, n = 7 *Aotus nancymaae* splenectomized monkeys were infected with *P. vivax* cryopreserved strains (one strain per animal) via intravenous injection following saline washes. Briefly, *P. vivax*-infected red blood cells (iRBC) cryopreserved stocks (approximately 1–2 million of parasites per stock) in glycerolite were thawed with 12% NaCl saline solution and incubated for 5 min at room temperature. After centrifuging for 4 min at 830×*g* the supernatant was discarded and iRBC was washed with 1.6% NaCl saline solution and centrifuged for 4 min at 830×*g*. Finally, iRBC were washed twice in 0.9% NaCl saline with centrifugation for 4 min at 830×*g*. The pellet was resuspended in 1 ml of RMPI 1640 for intravenous infection using the femoral vein of *Aotus nancymaae* NHPs, during this process we used ketamine–acepromazine mix for sedation. We performed parasitemia follow-up every other day from Day 3 to 42 using Giemsa-stained thin smear microscopy.

The same methods were used for experiments testing doses of the higher virulent *P. vivax* strain in *Aotus nancymaae* intact-spleen immunocompetent animals. First, we thawed a freezing stock to infect donor intact-spleen Aotus NHPs. Second, we collected fresh infected blood during peak parasitemia (days 10–14 and parasitemia range of 5000–50000 par/µl) and adjusted for infectious doses of 0.3 (n = 3), 1.0 (n = 6) and 2.5 (n = 3) × 10^6 iRBC. We performed follow-up every other day from Day 4 to 37 using Giemsa-stained thin smear microscopy. For all experiments, we calculated parasitemia/µl by counting number of infected red blood cells (RBC) in a total of 50 fields with ~ 200 RBC each and using proportion of infected RBCs with % of hematocrit for a final value, similar to our previous *P. falciparum* Aotus trials [17–19]. In experiments with evaluation of hematological parameters, 250 µL of peripherical whole blood was analyzed by a VetScan HM5 hematology analyzer. Hematological markers were adjusted using values derived from *Aotus nancymaae* colony in Peru. All quantitative data was validated by internal machine parameters identified by VetScan software.

### Evaluation of IgG antibody response against blood stage *P. vivax* MSP-1 antigen

For the evaluation of natural IgG response, we compared 1.0 (n = 6) vs 2.5 (n = 3) × 10^6 iRBC. An in-house ELISA was optimized for detection of IgG levels post-infection with the *P. vivax* Vietnam-IV strain. Briefly, 96-well microplates (Nunc Maxisorp) were coated with 4 μg/ml of *P. vivax* MSP-1 protein (bei resources, Cat#: MRA-47) and incubated overnight at 4Cº. The next day, plates were washed 5 times with PBS 1X/0.05%Tween 20, then plates were blocked for 1 h with TBS1X-Skin milk (SM) and washed. Monkey plasma samples were then added at 1:100 in TBS1X-SM and incubated for 2 h at room temperature. Antibodies were detected using peroxidase-conjugated anti-monkey IgG (dilution 1:1000, Sigma Aldrich Cat#: A2054) with an incubation of 1 h. O-phenylenediamine (Sigma Aldrich Cat#: P3804) with hydrogen peroxide used as substrate. The reaction was stopped after 1 h with 3N HCl, then plates were read at 492 nm. Optical density values were used to measure kinetics of IgG antibodies [38].

### Genomic analysis of *P. vivax* Vietnam-IV and characterization of DBP gene

A whole blood sample from an *Aotus nancymaae* NHPs infected with *P. vivax* Vietnam-IV was collected and used for DNA extraction using the QIAamp DNA Mini kit (Qiagen, Hilden, Germany) following the manufacturer’s protocol. Selective whole genome amplification was performed in two amplification rounds using 50 ng of gDNA and 50 μl of reaction as previously described [39]. Finally, the amplified genomic product was sequenced with both Illumina HiSeq and Oxford Nanopore MinION sequencing at Oregon Health & Science University and NAMRU SOUTH, respectively.

Short reads were filtered using Trimmomatic v0.36 with a minimum quality cutoff of 25 and a minimum length of 70 base pairs (bp). Long reads were filtered using Guppy v4 with parameters set to ‘High-accuracy base calling’ and ‘min_qscore = 7’ for base call model and read filtering. Short reads were aligned onto the reference Anan_2.0 *Aotus nancymaae* sequence with BWA-mem. Non-aligned short reads and nanopore long reads were used for a de novo hybrid alignment on SPAdes v3.13.1, contigs were blasted against NCBI and non-malaria contigs were eliminated, minimap2 v2.26 and Pilon v1.24 software were used for gap closing and error correction. Finally, scaffolding was performed onto the reference sequences PlasmoDB-46_*P. vivax* P01 strain using RagTag v2.1.0. [40–43].

Single nucleotide variants (SNVs) were jointly called using GATK V4.1.4.0 using a mapping quality score greater than 40.0 and quality by depth greater than 2.0. Subsequently, genetic polymorphisms were explored in vaccine candidate antigen genes with SNPEff. Polymorphic regions were extracted and used for gene ontology (GO) analysis on PlasmoDB using Bonferroni correction for multiple testing. Finally, DBP-II copy number for only Vietnam-IV strain (used for challenge model) was calculated from the read median depth in the gene corrected by the median depth of the chromosome.

### Production of mAb 099100 against *P. vivax* DBP-II protein

Previously, we isolated single anti *P. vivax* DBP-II-specific IgG + memory B cells from human individuals with high blocking in vitro activity in short-term culture [44]. We identified a panel of 12 DBP-II-specific human monoclonal antibodies (mAbs) from distinct lineages, which blocked DBP-II and red blood cell receptor DARC binding. We selected mAb 099100 for use in in vivo* Aotus nancymaae* model based in previous high efficacy score [44]. Briefly, two plasmids that included coding sequences for full-length IgG1 heavy and light chains were transfected into HEK293-H cells. Transfected cells were adapted for growth in Freestyle 293 serum-free expression medium under suspension conditions of 37 °C and 8.5% CO2. Cells were centrifuged post-transfection and culture medium was harvested, filtered through a 0.22 μm filter and supernatants concentrated 20 times using a 50 kDa cut-off Vivaflow 50 System. One volume of IgG binding buffer (Thermo Scientific Pierce) was added, and IgG was purified on a mAbSelect PrismA (Cytiva) eluted with IgG elution buffer pH 2.7 and neutralized with 1 M Tris pH 9.0. Resulting mAbs were concentrated and buffer exchanged with PBS using Amicon Ultra4 10 kDa and concentrations were determined on Nanodrop (Thermo Fisher Scientific) confirming purity by SDS-PAGE. In addition, levels of lipopolysaccharide (LPS) have been reduced during purification of mAb 099100 using a wash step with 30% isopropanol. Finally, mAbs were diluted in 1X PBS pH 7.2 and filtered (0.22 μm) prior to aliquoting and flash freezing in liquid nitrogen for transport to NAMRU SOUTH. The initial stock concentration of mAb was 160 mg/ml and was subsequently diluted in PBS 1X to final dose of 100 mg/Kg per each *Aotus nancymaae*.

### Evaluation of in vivo efficacy of mAb 099100 against *P. vivax *DBP-II protein in *Aotus nancymaae* blood stage model

Recently, subcutaneous administration at doses of 150–300 mg of antimalarial monoclonal antibody CSP-L9LS against *P. falciparum* infection showed an efficacy of 66–70% in a field trial in children [45]. These data encouraged the use of monoclonal antibodies against malaria infection produced by *P. falciparum* or other malaria species.

We administered mAb 099100 subcutaneously three days before challenge at a concentration of 100 mg/Kg. At day of challenge, monkeys were infected with fresh blood from a donor NHP at dose of 2.5 × 10^6 *P. vivax* Vietnam-IV iRBCs via intravenous route as above. All NHPs were followed up by microscopy as described above every other day during Day 3 to 35 post-challenge with efficacy determined by day of pre-patency positive parasitemia or treatment need. Treatment end points included parasitemia > 250,000 par/µL in peripheral blood, a hematocrit below 25% or platelets (Plt) below 20,000/mm^3 (very severe thrombocytopenia). Single dose mefloquine (MQ) treatment of 25 mg/Kg was initiated when monkeys reached treatment end points.

### Antibody kinetics

Kinetics of human mAbs 099100 against DBP-II (at days 0, 7, 15, 21 and 35) and VRC-01 against HIV (at Day 0, 7, 21 and 35) proteins were tested by ELISA using *Aotus nancymaae* serum samples. Briefly, DBP-II and HIV proteins were coated in 96-well microplates (Nunc Maxisorp) at a concentration of 1 μg/ml diluted in PBS 1X buffer and incubated overnight at room temperature. The next day, plates were washed with PBS 1X/2% Triton X-100 then blocked for 1 h with blocking buffer (PBS 1X with 1% BSA / 5% of skim milk) and washed again three times. Serum samples were added in a three-fold serial dilution starting at 1:10. A standard curve of each mAb was used with serial dilution of 1:3 starting at a concentration of 10 ug/ml. After a 2 h incubation, the plates were washed 3 times and anti-human IgG (Jackson ImmunoResearch Labs Cat #: 709-036-098) was added at a dilution of 1/10,000 and incubated for 1 h at room temperature. After washing three times, TMB substrate solution (Thermo Scientific, Cat:# 34018) was added and incubated for 5 min at room temperature. Finally, we added Hydrochloric acid [3N] as stop solution and read at 450 nm in a Biotek ELISA reader. Values were expressed in optical density (OD) and antibody concentration was analyzed in Graph Pad using Log OD values and nonlinear regression (curve fit) of samples versus standard curve. Finally, function of log (agonist) vs response- variable slope (four parameters) was used to calculate EC50 values of mAb for each sample tested.

### Statistical analysis

Analysis was performed using STATA v16.0 statistical software (Stata Corp., College Station, TX, USA) and GraphPad Prism v9 (GraphPad Software, LLC). Differences among the frequency of immunological variables were analyzed using Chi-square test for categorical variables and Mann–Whitney or Kruskal–Wallis test for numerical continuous variables to compare the median values.

Efficacy of each mAb against DBP-II was assessed using Kaplan–Meier survival curves and log rank test for microscopy patency and treatment end point. Significance for all statistical analysis was set at *p* < *0.05* or *p* < *0.001.*

## Results

### *P. vivax* Vietnam-IV results in predictable and robust infection

**Fig. 1:**
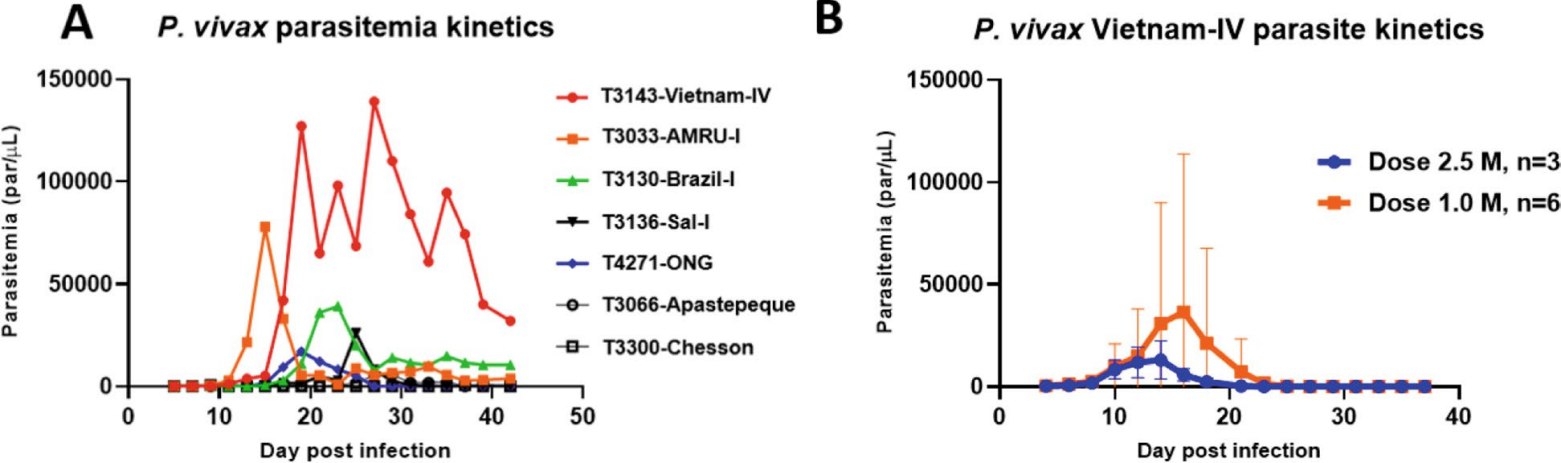
*P. vivax* Vietnam-IV produces robust and consistent infection kinetics. **A** Parasitemia kinetics in splenectomized monkeys, each color line represents parasitemia kinetics by each *P. vivax* monkey adapted strain (n = 1/strain). **B** Parasitemia in spleen-intact NHPs, where each color line represents average of parasitemia kinetics of *P. vivax* Vietnam-IV at a dose of 2.5 × 10^6 iRBC (blue line) and 1.0 × 10^6 iRBC (orange line). Standard deviation whiskers are showed during all parasitemia kinetics at two doses of *P vivax* Vietnam-IV

Currently, few *P. vivax* strains have been evaluated in NHP models for malaria blood stage research. Collins et al. during 1970-90 s tested *P. vivax* strains collected worldwide and a number demonstrated infection capacity in *Aotus*. In an attempt to develop a robust *Aotus* animal model for *P. vivax* blood stage research similar to the *Pf* blood stage challenge in *Aotus*, we first tested seven different *P. vivax* strains for their ability to infect splenectomized *A. nancymaae* NHPs. During 42 days of follow-up, we observed variable parasitemia kinetics between strains with one strain (Vietnam-IV) showing a higher parasitemia with multiple peaks (between 98,000–139,000 par/µl) and longer periods of active parasitemia compared with the others (Fig. 1A).

Based on this, we selected Vietnam-IV to move forward as a potential blood stage challenge strain. For this, we tested two doses in a minimal number of immunocompetent intact-spleen monkeys to provide data on a dose gave that would give the best infection kinetics for testing interventions in the minimum number of animals. A dose of 1.0 × 10^6 iRBC (n = 6) as well as a smaller pilot dose of 2.5 × 10^6 iRBC (n = 3) was tested in the first experiment.

The dose of 1.0 × 10^6 had parasitemia peaks between 2100–194,000 par/µL at day 13 ± 2, with pre-patency (first day of positive parasitemia) period of 5 ± 1 days (Fig. 1B). Parasitemia kinetics of 2.5 × 10^6 iRBC dose showed a minimum and maximum parasitemia of 13,000–21,600 (mean 16,400 ± 3,735 par/µl) with a pre-patency parasitemia at day 5 ± 1, and peak parasitemia at day 13 ± 2. The pre-patent period and day of peak parasitemia were similar to our previous data using *P. falciparum* blood stage challenge in *Aotus nancymaae* animals [14, 16]. The higher dose showed lower overall variation, with a lower coefficient variation for day of peak parasitemia (15% vs 17%) and maximum parasitemia (23% vs 164%). All monkeys self-cure parasitemia in both groups.

### IgG antibody response against *P. vivax* MSP-1 during blood stage

**Fig. 2:**
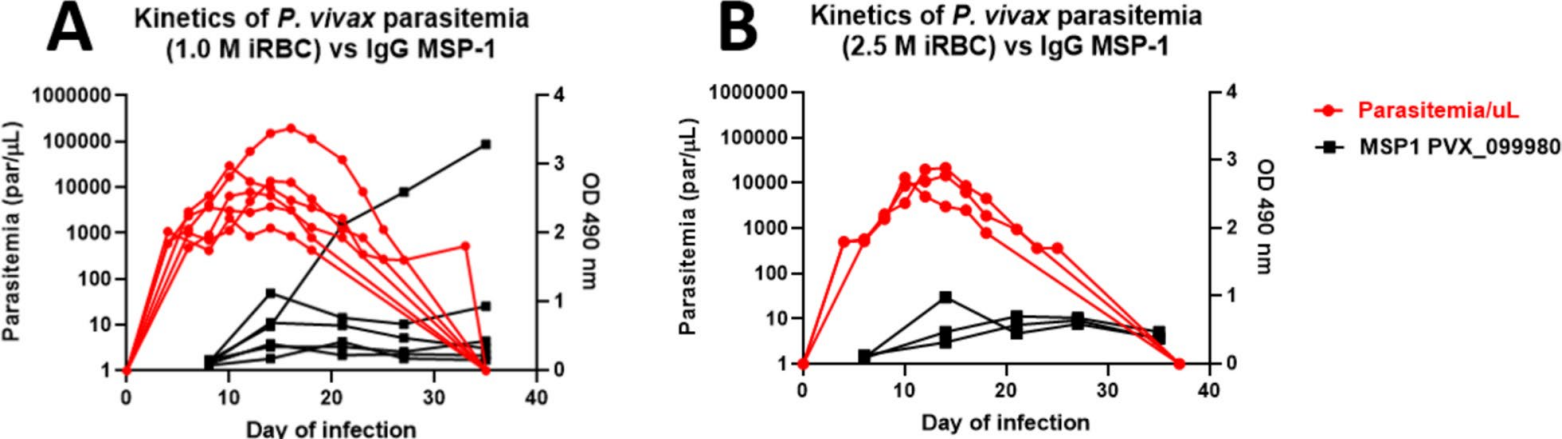
Kinetics of average values of *P. vivax* parasitemia/ul in blood (red lines) vs antibodies levels in *Aotus nancymaae* monkeys measured by OD values (black lines) against *P. vivax* MSP-1 antigen post infection with 1.0 × 10^6 iRBC (n = 6) or 2.5 × 10^6 iRBC (n = 3) infectious doses in A or B, respectively. Both *Aotus nancymaae* group showed self-cure parasitemia at the end of follow-up of 35 days for IgG kinetics evaluation

In order to gain insights into the antibody response to a single infection, we measure IgG to the well-studied and highly immunogenic protein MSP-1 (then cite your human paper).Plasma samples from the above groups were used for serological analysis. All Aotus NHPs from the 2.5 × 10^6 iRBC and 1.0 × 10^6 iRBC dose groups showed positive natural IgG antibodies reactivity against the blood stage protein MSP-1 (Fig. 2). We observed a peak of IgG antibodies between days 14–21, post-peak parasitemia for both groups. Acknowledgment of the kinetics of naturally acquired IgG immunity post-malaria infection could be useful to identify any potential interference during efficacy testing of novel vaccines and mAbs.

### Measuring hematological parameters and dose sensitivity of *Pv* Vietnam-IV infection

**Fig. 3:**
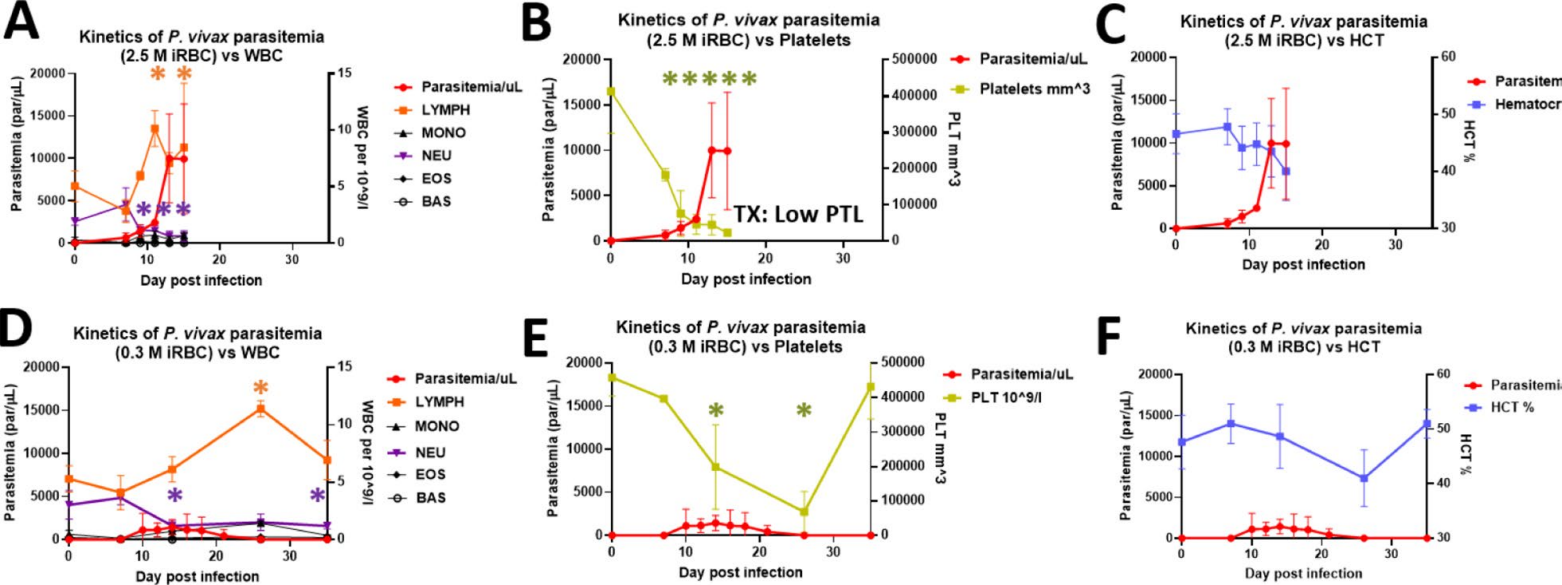
Evaluation of hematological markers (White blood cells, platelets and hematocrit %) post infection with *P. vivax* Vietnam-IV at high dose of 2.5 × 10^6, n = 3 (**A**–**C**) and low dose of 0.3 × 10^6, n = 3 (**D**–**F**). TX: malaria treatment, PTL: Platelets * for p < 0.05. Only Aotus monkey from 2.5 M iRBC group were treated for low PLT

We next wanted to assess the reproducibility of the 2.5 × 10^6^ dose (given the lower variation coefficient during parasitemia follow-up) as well as pilot a lower dose. In addition, we evaluated hematological markers during infection as an additional potential readout of disease. Our results showed variable kinetics of WBC, with significant increase of lymphocytes (5.0–5.3 to 7.0–8.5 10^9/l), decrease of neutrophils (1.9–3.0 to 0.6–1.2 10^9/l) and platelets (413,000–458,000 to 23,000–68,000 mm^3) during parasitemia **(**Fig. 3)**.** The higher dose showed a faster change of hematological values compared with the low dose, with platelets markedly decreasing as early as day 7 in the high dose group (lowest average values of platelets for low and high infectious dose were 68,000 ± 48,000 and 23,000 ± 8,000 mmm^3, respectively). Thrombocytopenia was previously observed during malaria infection in humans with values of < 50,000/mm^3 for severe and < 20,000/mm^3 for very severe [46–48]. Considering these values, we initiated treatment at < 20,000 mm^3 and maintained this as a treatment endpoint in subsequent experiments. In this experiment, all high-dose NHPs received malaria treatment due to severe thrombocytopenia. In contrast, Aotus NHPs from the low dose showed a self-progressive recovery to normal values at day 28 post-infection **(**Fig. 3)**.** Considering the above data, *P. vivax* Vietnam IV appears to provide consistent infection parameters in spleen-intact *Aotus nancymaae* animals. Going forward, we chose a dose of 2.5 M iRBCs as this showed higher and more consistent infection parameters than 0.3 or 1 M, with additional marker of disease (thrombocytopenia).

### Sequencing analysis of *P. vivax* Vietnam-IV and characterization of DBP gene

Genomic analysis of malaria parasites is necessary to identify mutations in critical immunogenic targets, which can affect the design of malaria vaccines. Currently, few monkey *P. vivax* adapted strains are fully characterized and publicly available, with only SAL-I and AMRU-I monkey-adapted strains sequenced by selective whole-genome amplification (sWGA) [49].

Genomic analysis of the Vietnam-IV strain compared with reference strain PvP01, showed that nucleotide variants were found in 5,606 coding regions with high, moderate, low, or modifier impact which are associated with loss of function, nonsynonymous variant without loss of function, synonymous variant and non-coding variants, respectively (Supplementary data, Table 1). Of all the variants, 187 were of high impact and 24,282 of moderate impact, distributed in all 14 nuclear chromosomes (Supplementary data in Tables 2 and 3, respectively). On the other hand, GO of cellular components showed statistical significance enrichment on 25 GO nodes (Bonferroni-corrected p < 0.05) associated mainly with membrane and host cellular components (Supplementary data, Table 4). Finally, we compared genomic information between PVP01 and Vietnam-IV *P. vivax* strains to detect amino acid variation in immunogenic targets as CSP, SSP2/TRAP, CelTOS, MSP1 and others (Supplementary data, Table 5).

**Table 1:**
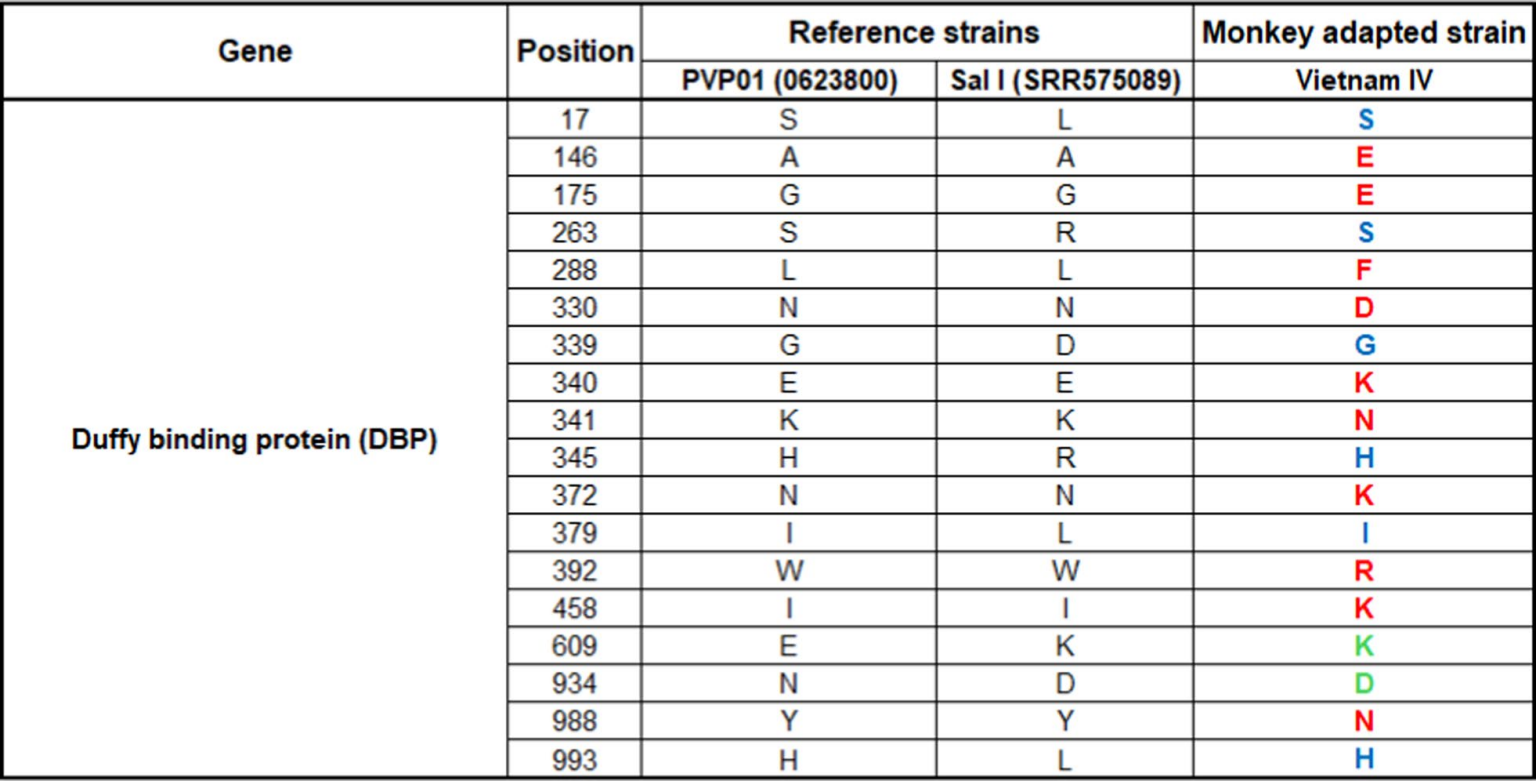
Analysis of amino acid changes in DBP gene comparing reference strains

*P. vivax* PVP01 and SAL I versus Vietnam-IV monkey adapted strain. Changes were evaluated in whole protein size of 1082 amino acids. Vietnam-IV amino acid changes with similar pattern with PVP01 or SAL I was labeled in blue or green color, respectively. Red color represents changes in amino acid different to PVP01 and SALI

We next performed more in-depth characterization of the gene encoding the most advanced blood stage vaccine candidate, DBP. We sequenced the whole DBP (1070 aa) [50]. The Vietnam-IV sequence showed a single copy, confirmed by the read median depth in the gene corrected by the median depth of the chromosome. We have validated the accuracy of the method considering genes with > 50% coverage and at least 15 × mean read depth. Using these parameters, we checked that well-described single copy genes such as chloroquine resistance transporter (crt-o), ookinete surface protein (Pvs25) and fructose-bisphosphate aldolase are accurately identified as single copy genes. In addition, to determine single clonality we conducted an analysis of the distribution of minor allele frequencies (MAF) in Vietnam-IV strain using high quality SNPs, limiting only positions with quality higher 30 and read depth higher than 20. The MAF distribution was mostly represented by a peak near 0 with very few SNPs with MAF > 0.05. This result is consistent with a monoclonal sample or the presence of a dominant clone.

Finally, we compared amino acid sequences of DBP from *P. vivax* Vietnam-IV versus *P. vivax* PVP01 (Pv_0623800) and SAL I (SRR575089) reference strains to determine specific mutations between strains. Eighteen amino acid changes in Vietnam-IV were found, showing more similarities with PVP01 which was isolated from Indonesia. None of these mutations were related to binding size of published anti-DBP-II monoclonal antibodies.

### Pilot passive transfer of anti-*Pv *DBP-II mAb 099100 followed by *Pv* Vietnam-IV blood stage challenge

**Fig. 4:**
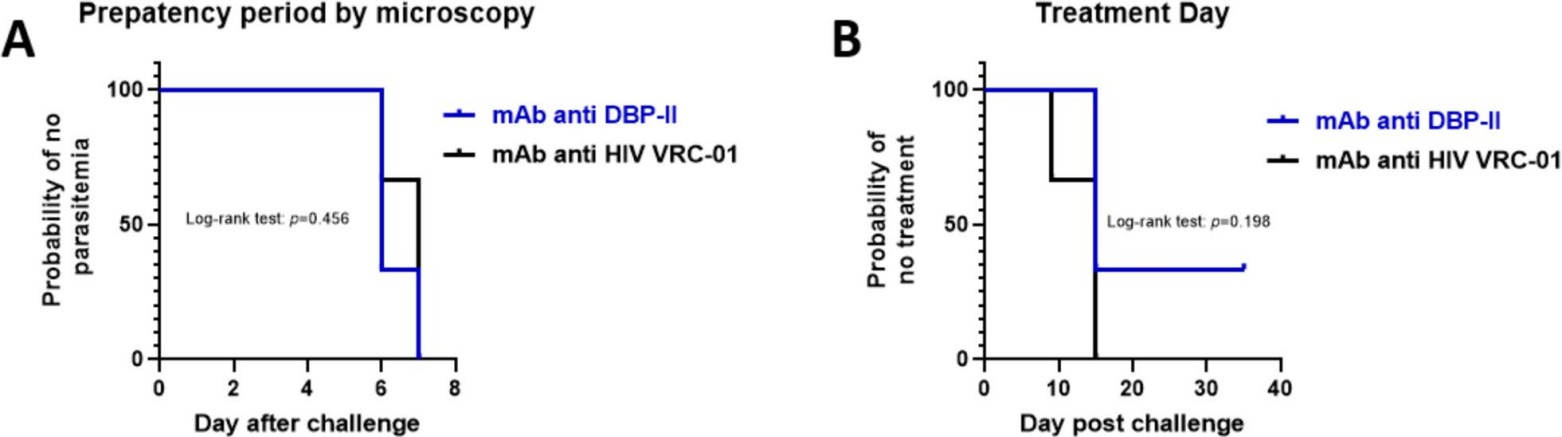
Kaplan–Meier analysis of prepatency parasitemia (**A**) and treatment day (**B**) were evaluated in Aotus NHPs challenged with 2.5 × 10^6 *Pv* Vietnam IV iRBCs. Blue and black lines represent Aotus monkeys from mAbs against DBP-II and HIV-VRC01-LS groups, respectively. Significant difference between groups were represented by *p < 0.05 and **p < 0.001

To test *Pv* Vietnam-IV infection of *A. nancymaae* NHPs as a challenge model, we performed a pilot study using the anti-*Pv* DBPII mAb 099100, which demonstrated in vitro efficacy against short term *Pv* blood stage culture and *Pv*DBP*-*transgenic *P. knowlesi *in vitro growth inhibition with an IC_50_ of 135 μg/mL [51]. Animals (n = 3/group) were administered 100 mg/kg of 099100 or mAb anti-HIV VRC01-LS (control) 3 days before infection with 2.5 × 10^6^ *Pv* Vietnam-IV iRBCs. Sterile protection, defined as negative parasitemia throughout follow-up, was not achieved in any animal. Instead, all animals had positive parasitemia at Day 7 with no significant variation for onset of parasitemia (log-rank test p = 0.456) (Fig. 4A). A secondary measure was reaching a pre-defined treatment endpoint based on parasitemia or hematological parameters. In this regard, all monkeys (3/3) from the mAb anti-HIV VRC01-LS group were treated (one at day 9 post-challenge, Plt:6,000 mm^3 and two at day 15 Plt: 15,000 and 20,000 mm^3) compared with the mAb 099100 group where 2 of 3 Aotus NHPs were treated (both NHPs at day 15 post-challenge, Plt: 18,000 and 19,000 mm^3) (Fig. 4B). The one untreated monkey had no significant decrease of platelets during parasitemia peak (Day 9: 1,765 par/μL and Plt: 247,000 mm^3). However, no significant difference was observed between both groups for the day of treatment following infection (Kruskal–Wallis test treatment day p = 0.198).

**Fig. 5:**
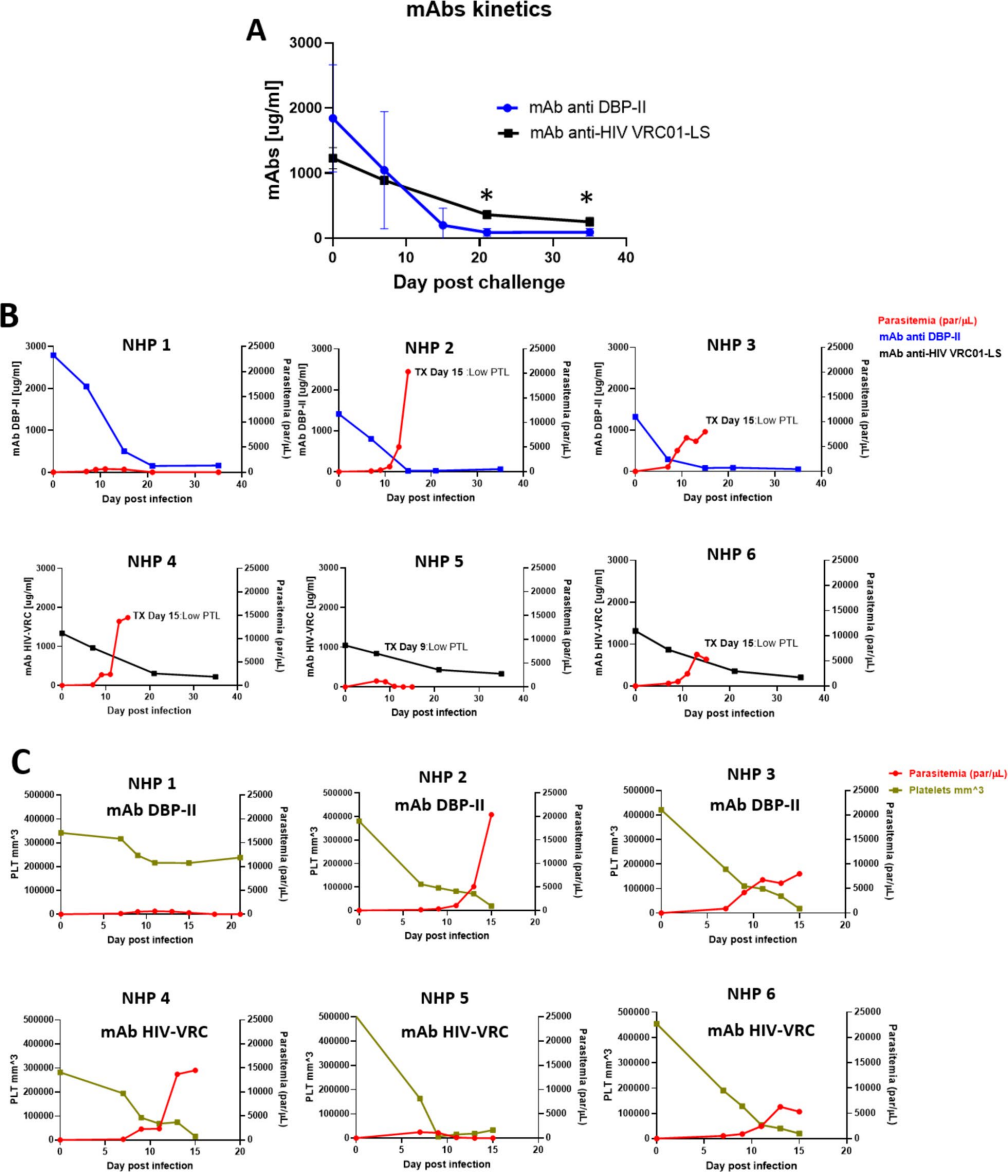
Kinetics of platelets and parasitemia following passive transfer and challenge. **A** Average values of mAbs kinetics of DBP-II (n = 3, blue lines) and VRC01-LS (n = 3, black lines). **B** Individual values of mAb kinetics vs parasitemia (red lines) post challenge in DBP-II and VRC-01-LS groups. TX: monkey treated by low platelets levels (< 20,000 mm^3). **C** Individual values of platelets kinetics vs parasitemia (red lines) post challenge in DBP-II and VRC-01-LS groups. TX: monkey treated by low platelets levels (< 20,000 mm^3), PTL: Platelets. NHP: non-human primate. Significant difference between groups were represented by *p < 0.05 and **p < 0.001

Evaluation of mAbs kinetics showed that the average mAb concentration at day of challenge (3 days after subcutaneous mAbs administration) were 1,843 ± 672 ug/ml (half-life average: 6 days) and 1,232 ± 132 ug/ml (half-life average: 12) for DBP-II 099100 and anti-HIV VRC01-LS, respectively (Fig. 5A). Concentration of mAbs between groups was similar during the day of challenge and day 7, with significantly lower values during days 21–35 for mAbs DBP-II compared with anti-HIV VRC01-LS. Individual screening of mAb kinetics for both groups versus parasitemia showed that NHP #1 had a higher amount of anti-DBP-II mAb at Day 0 of 2,792 ug/ml compared with other monkeys within the group (Fig. 5B) and lowest parasitemia level and did not require treatment.

## Discussion

Currently, progress in developing *P. vivax* blood stage vaccines and immunoprophylactics is constrained by a lack of tractable in vitro and in vivo models. This limitation has affected the pipeline of new vaccine candidates, with DBP-II the only blood-stage vaccine candidate in Phase I. Developing novel animal models is essential to speed up the creation and selection of the next generation of *P. vivax* blood stage vaccines and immunoprophylactics. This study introduces a preliminary development of *P. vivax* blood stage challenge model that could bridge a critical gap in the development process for *P. vivax* blood stage interventions.

Globally, few accredited centers have the expertise and capacity to perform pre-clinical malaria studies using *Aotus* NHPs. The Gorgas Memorial Laboratory in Panama has been working for decades on evaluating malaria biology, antimalarial chemotherapy, and efficacy of vaccines against blood stage malaria using *Aotus lemurinus* [52]*.* However, limited information is available regarding susceptibility of infection with multiple *P. vivax* strains, deep characterization of parasitemia during the course of infection, and the subsequent changes in hematological values in *Aotus nancymaae* monkeys with intact spleens.

Here, we showed that colony-bred *Aotus nancymaae* monkeys from the Peruvian Amazon Basin region are susceptible to infection using *P. vivax* strains that were previously adapted by Collins et al. with origins from Asia (Vietnam-IV, AMRU-I, ONG, and Chesson) and the Americas (Brazil-I, Sal-I and Apastepeque) [53–56]. Few previous NHPs models evaluated susceptibility across multiple *P. vivax* strains. Instead, these models typically focused on one or two strains at the same experiment [49]. Thus, our study is unique in evaluating multiple *P. vivax* strains in a single animal species readily available in multiple research facilities accredited by the Association for Assessment and Accreditation of Laboratory Animal Care (AAALAC). Studies that build off these findings will be necessary for better understanding *P. vivax* malaria biology and host restriction factors.

The parasitemia in our limited number of NHPs showed that *P. vivax* Vietnam-IV is highly virulent compared with the other *P. vivax* tested strains. Our results align with a previous study where a *P. vivax* strain from Vietnam was more virulent than strains from Asia or the Americas [55]. In addition, the high infectious dose of 2.5 × 10^6 iRBC showed lower variation in parasitemia kinetics compared with lower doses. An animal model with low variation is critical to reduce the number of animals needed for definitive outcomes. The correlation between infective dose and low variability has also been seen in a *P. knowlesi* sporozoite infection model in rhesus NHPs [57].

*P. vivax* blood stage infection with Vietnam-IV strain in *Aotus nancymaae* NHPs showed significant changes in hematological values. Lymphocyte dynamics significantly increased during peak parasitemia in *Aotus nancymaae* NHPs, which contrasts with lymphocyte dynamics in humans where lymphopenia is described in CHMI or during natural infection [33, 58]. The mechanism involved in human lymphopenia is hypothesized to be related to lymph node sequestration, parasite-induced cell death or interaction of immune check points (inhibitory molecule PD-1 in Treg cells) [59]. Thus, this incongruence should be further explored in future studies of *P. vivax*-infected *Aotus* NHPs. Thrombocytopenia and neutropenia were associated with *P. vivax* parasitemia kinetics in *Aotus* NHPs, similar to human populations during severe malaria disease. In humans, severe thrombocytopenia was identified in children or adult populations as a marker for increased risk of death during *P. vivax* malaria, particularly in subjects with severe anemia [47, 60, 61]. Here, we presented severe thrombocytopenia as a novel treatment endpoint in our *Aotus nancymaae* model. While our animals developed hematological signs of disease at a greater frequency than humans, this consistency could be exploited to assess the effect of novel *P. vivax* interventions and to understand the biology of the parasite better.

Few *P. vivax* monkey adapted strains, such as AMRU-I and Sal-I, have been characterized at the genome level. Here, we also provided new genomic information that Vietnam-IV is clonal and has only one copy Pv DBP. A number of *P. vivax* isolates have been identified with Pv DBP amplification hypothesized to be involved in evasion of host immunity, or alternative invasion pathways [62, 63]. Our well characterized genomic data of *P. vivax* Vietnam-IV in region II of the Pv DBP that contains the binding motif to DARC will be important in understanding the efficacy *P. vivax* DBP vaccines and prophylaxis or therapeutic monoclonal antibodies target in the *Aotus* model. However, additional sequencing is needed to provide a high-quality whole genome coverage for this strain.

The recent development of prophylactic monoclonal antibodies for malaria has been highly successful, with mAbs targeting PfCSP providing up to 88% efficacy against clinical diseases in the endemic population [57]. There are only a limited number of mAbs in NHPs for blocking blood stage malaria infection. Two leading candidates are PvDBPII and PfRh5, two essential invasion ligands for *P. vivax* and *P. falciparum* blood stage infection, respectively. Here we tested a human mAb to Pv DBPII (mAb 099100) that has an in vitro IC_50_ of 135 ug/ml using PkPv DBPII transgenic parasite with the SAL I variant of Pv DBPII [51]. Monoclonal antibody 099100 showed about 50% blocking of multiple *P. vivax* clinical isolates in vitro and variation in blocking was not related to differences in polymorphisms in Pv DBPII but only for Pb DBPII copy number with more copies resulting in less inhibition [63]. Passive transfer of mAb 099100 provided little protection against *Pv* Vietnam IV challenge, even though serum mAb levels were > 1000 ug/ml at the time of challenge, well above IC_90_ of 553 ug/ml [51]. There are two possible explanations. First, polymorphisms might be present at the mAb binding site of mAb 099100. This mAb was not crystallized, so the exact binding regions are unknown. However, two other blocking mAbs 053054 and 092096 [64] were crystallized and competition experiments show that they shared or had overlapping epitopes [44]. The epitopes are in domain 2 of PvDBPII, compressing residues Y219, E249, L270-K289 and Q356-W375, based on the SALI sequence [64]. Vietnam IV has 3 residues that differ from SAL-I variant in or near this epitope, R263S, L288F and N372K. Whether these differences influence the mAb efficacy is unclear. A second possibility is that insufficient levels of mAb were sustained during infection. This was observed with a previous *Aotus* study conducted in our facility testing the *Pf*Rh5 mAbs where additional boosters at Days 7 and 14 were used to maintain protective levels mAb [19]. In this regard, our data suggests that low efficacy of mAb DBP-II 099100 could be potentially due to a quick decrease of mAbs concentration (1,843 ± 672 µg/mL at day 3 versus to 90 ± 51 µg/mL at day 21). Future studies should prioritize experiments to determine if mAbs require greater potency or sustained concentrations through repeated administration or extended half-lives. The potential of the latter was shown here as the HIV-VRC01 mAb had the same IgG1 backbone as mAb099100 but also had a mutation in the Fc region at M428L and N434S referred to as LS, which extended the antibody half-life. These studies thus lay the groundwork for developing the next generation of highly protective mAbs against *Pv* infection [45].

Our study has several limitations. Specifically, we used n = 1 splenectomized animals for the initial down-selection of viable strains. We used the *P. falciparum* FVO strain as an example of a blood stage challenge parasite which produces robust and consistent infection in 100% of *Aotus nancymaae* regardless of splenectomy. With this high bar, n = 1 is the minimum number of animals needed to observe infectivity of an FVO-like strain of *P. vivax*, and this appears to have been achieved with Vietnam IV. Still, it is possible that the other strains would have produced similarly robust infections after further passages and adaptations. This will be useful in assessing new interventions for efficacy against multiple strains. There was also shortage of serum samples that prevented us from performing analysis of hematology markers and antibody kinetics in the initial infection experiment. Due to their small size, blood draws from *Aotus nancymaae* NHPs are limited based on weight and periodicity of sampling which is a major consideration and limitation of their use. Other potential limitations of the Aotus model include the required permitting from local (SERFOR) and external institutions such as Convention on International Trade in Endangered Species of Wild Fauna and Flora (CITES) for sample sharing internationally. In addition, Aotus are a non-natural host for P. vivax which is likely to impact biological relevance. However, this is also true for *P. falciparum* in Aotus and *P. cynomolgi* in rhesus macaques which are commonly used and in the case of Rh5 has predicted human vaccine results. Finally, in our passive immunity transfer study that served as a pilot for testing new interventions, the only animal with high anti-DBPII mAb titers was protected from high parasitemia and thrombocytopenia, and was the only animal that did not require treatment. However, in light of the statistical insignificance and the fact that one NHP in the control group also failed to develop parasitemia in the 5,000–15,000 par/μL range that was seen in other control animals, any biological connections will require larger sample sizes. In general, there was a lack of correlation between thrombocytopenia and parasitemia which is also observed in humans [60]. This and apparent variability in parasitemia will be important when considering endpoints in future studies that do not rely solely on preventing infection. Finally, a critical limitation of this pilot efficacy study is the potential confounding role of anti-drug antibodies (ADA) elicited against the non-simianized human DBP-II mAb. While sample volume constraints precluded a formal retrospective ADA analysis, the rapid clearance of DBP-II is consistent with the intrinsic pharmacokinetic limitations of unmodified human IgG1 in the Aotus model, as similarly reported for chimeric mAbs lacking half-life extension modifications [19]. This is further supported by our internal control, VRC01-LS, which demonstrated sustained durability attributable to the 'LS' mutation in its Fc domain, a modification absent in DBP-II. Consequently, we strongly recommend that future mAb efficacy evaluations in Aotus utilize fully simianized constructs or incorporate Fc modifications (e.g., LS) to extend half-life, coupled with prospectively validated ADA monitoring protocols to definitively decouple intrinsic clearance from host immune rejection.

In conclusion, we demonstrated that the *A. nancymaae* NHPs model provides a *P. vivax* blood stage infection model when challenged with a defined infectious dose of the Vietnam-IV strain for which we provide genomic data. The infection and need for treatment in 100% of animals enables powering of studies where proportion of animals infected or treated is the primary endpoint. Studies that rely on kinetics will be more impacted by the apparent inter-experiment variability, but the data presented here can be used to perform power analyses for appropriate sample sizes of future studies. This model can become a valuable resource for evaluating the efficacy of the next generation of antimalarial drugs, vaccines, and immunoprophylactics against *P. vivax* blood stage infection.

## Supplementary Information

Supplementary material 1.

Supplementary material 2.

## Data Availability

No datasets were generated or analysed during the current study.
