# Supplementary material for: Development of novel Aotus nancymaae non-human primate model for the evaluation of Plasmodium vivax blood stage vaccines and immunoprophylactics
Source: Malar J. 2026 Jun 3;25:290. doi: 10.1186/s12936-026-05960-7 (PMC13455277; doi:10.1186/s12936-026-05960-7)
Supplement: Supplementary file 1 [file 12936_2026_5960_MOESM2_ESM.docx]

**Supplementary data**

**Production of mAb 099100 against *P. vivax* DBP-II protein**

We produced successfully mAb 099100 (red line) with a molecular weight of ~185 kDa, likely due to glycosylation. No aggregates were detected, and the fraction of potential dimer is less <1% as showed by spectrometry.

**Evaluation of potential interference of natural response during administration of mAb DBP-II**

We measured the kinetics of natural antibody response against DBP-II region. We used the VRC01 LS group avowing the cross reactivity of mAbs anti DBP-II. Our results showed a minimal potential interference of natural antibodies against DBPII region post challenge with *P. vivax* Vietnam-IV.

**Evaluation of clonality by minor allele frequency analysis**

We determined single clonality performing analysis of the distribution of minor allele frequencies (MAF) in Vietnam-IV strain using high quality SNPs. The MAF distribution was mostly represented by a peak near 0 with very few SNPs with MAF >0.05. Vietnam-Iv strain showed a monoclonal sample or the presence of a dominant clone for this study.
